# Combined Analysis of Untargeted Metabolomics and Transcriptomics Revealed Seed Germination and Seedling Establishment in *Zelkova schneideriana*

**DOI:** 10.3390/genes15040488

**Published:** 2024-04-12

**Authors:** Fengxia Yan, Tangmei Wei, Chao Yang, Yanbing Yang, Zaiqi Luo, Yunli Jiang

**Affiliations:** 1 Key Laboratory of National Forestry and Grassland Administration on Biodiversity Conservation in Karst Mountainous Areas of Southwestern China, Guizhou Academy of Forestry, Guiyang 550005, China; yfx19871017@163.com (F.Y.); yangyanbingjh@163.com (Y.Y.); 13984188836@163.com (Y.J.); 2 Xingyi Forestry Bureau, Qianxinan Prefecture Guizhou, Guiyang 562400, China; zhouzx2016@163.com; 3 Institute for Forest Resources and Environment, Guizhou University, Guiyang 550025, China; chaoyang202006@163.com

**Keywords:** *Zelkova schneideriana*, transcriptomic, untargeted metabolomics, seed germination, seedling establishment

## Abstract

*Zelkova schneideriana* Hand.-Mazz is a valuable ornamental tree and timber source, whose seedling breeding and large-scale cultivation are restricted by low seed germination and seedling rates. The regulatory mechanisms underlying seed germination and seedling establishment in *Z. schneideriana* remain unknown. This study conducted metabolomic and transcriptomic analyses of seed germination and seedling establishment in *Z. schneideriana*. Regular expression of genes and metabolite levels has been observed in plant hormone signal transduction, starch and sucrose metabolism, linoleic acid metabolism, and phenylpropanoid biosynthesis. The reduction in abscisic acid during seed germination may lead to seed release from dormancy. After the seed is released from dormancy, the metabolic levels of auxin, cytokinins, brassinolide, and various sugars are elevated, and they are consumed in large quantities during the seedling establishment stage. Linoleic acid metabolism is gradually activated during seedling establishment. Transcriptome analysis showed that a large number of genes in different metabolic pathways are upregulated during plant establishment, and material metabolism may be accelerated during seedling establishment. Genes regulating carbohydrate metabolism are altered during seed germination and seedling establishment, which may have altered the efficiency of carbohydrate utilization. In addition, the syntheses of lignin monomers and cellulose have different characteristics at different stages. These results provide new insights into the complex mechanisms underlying seed germination and seedling establishment in *Z. schneideriana* and other woody plants.

## 1. Introduction

Seed germination is a critical stage in the plant life cycle, representing the initial phase of plant growth and increasing yield [1]. It begins with seed imbibition and ends with radicle development [2]. Seedling establishment is another key stage of the plant life cycle that follows closely after germination. It is believed that well-developed seedlings result in well-developed plants [3]. Consequently, both seed germination and seedling establishment are essential for subsequent plant development. It is worth noting that both processes are powered by energy stored in the seed itself [4]. Seedling establishment has traditionally been studied as part of the germination process. However, several studies have indicated that these two consecutive steps are developmentally distinct and regulated by common and unique factors [5].

Plant hormones are crucial metabolites synthesized by plants to orchestrate growth and regulate and coordinate cellular division and differentiation, thereby governing seed dormancy, germination, and overall plant development [6,7]. ABA, GA, IAA, CTKs, and BR are pivotal regulators of numerous physiological and biochemical processes in plants [8], exhibiting distinct roles in seed germination and primary growth. Numerous studies have demonstrated pivotal regulatory roles of ABA and GA in seed dormancy and germination. The ABA level increased, whereas the GA concentration decreased in dormant mature seeds. ABA induces and maintains seed dormancy, whereas GA alleviates the inhibitory effect of ABA, thereby promoting seed germination [9,10]. Although there is no evidence supporting the importance of IAA in seed germination, it plays a crucial role in subsequent seedling establishment [11,12]. Furthermore, appropriate accumulation of IAA can facilitate the establishment and growth of seedlings, whereas excessive IAA levels are believed to impede this process [13]. BR has been shown to promote seed germination by modulating the inhibitory effect of ABA on this process and represents one of the core hormones that regulate plant growth [14,15,16]. CTK regulates all aspects of plant physiology and exerts its influence throughout all stages of seed germination and plant growth [17,18]. The types of material reserves present in plant seeds primarily include carbohydrates, proteins, and lipids [19]. Previous investigations have revealed that storage materials, such as starches, proteins, and lipids, are consumed during seed germination and seedling establishment [19,20,21]. Furthermore, these storage contents have also been found to affect both the rate and efficiency of seed germination [22]. To further elucidate the regulatory mechanisms underlying seed germination and seedling establishment, a comprehensive investigation of the dynamics of metabolite levels is imperative.

Several genes involved in the regulation of seed germination have been identified in different studies involving plant hormone signaling pathways [23], lipid metabolism, starch and sucrose metabolism [24], and phenylpropanoid metabolism [25]. In addition, ABA INSENSITIVE 3 (ABI3), FUSCA3 (FUS3), LEAFY COTYLEDON 1 (LEC1), and LEC2 are considered key transcription factors that specifically regulate seed dormancy in *Arabidopsis* [26,27,28]. At the same time, transcription factors such as LEC1, MYB, bHLH, and WRKY have also been confirmed to be involved in the seed germination process. Most of them are involved in regulating physiological activities mediated by hormone signals and play an important role in controlling embryo and endosperm development, regulating seed growth potential and delaying seed germination [29,30,31,32,33,34].

*Z. schneideriana* is a deciduous tree of the Ulmaceae family that is endemic to China. It is one of the major species widely planted in China because of its high-quality timber and high ornamental value [35]. Owing to habitat destruction and large-scale cut-down, the population size and distribution area of *Z. schneideriana* have shrunk [36]. Therefore, it was listed as one of the key protected wild plants of China in 1999 and has been ranked as a rare and endangered plant of second-grade protection in China since 2021. The International Union for Conservation of Nature (IUCN) includes this species in the Red List of Threatened Species [37]. Seed reproduction is the primary propagation mode. However, seedling breeding and large-scale cultivation of *Z. schneideriana* are seriously affected because of their low seed germination and seedling rates. At present, the mechanism of seed germination and the subsequent establishment of the plant after germination in *Z. schneideriana* is still unclear; a large part of the reason is that it is difficult to conduct in-depth research on *Z. schneideriana* using molecular genetic tools. Transcriptome and metabolomics is a systematic biological methodology, which is a powerful tool for explaining complex features. We attempted to use non-targeted metabolomics and transcriptomics techniques to study the dynamic changes in metabolites and gene expression during seed germination and seedling establishment in *Z. schneideriana*, to reveal the potential regulatory mechanisms in a tiny seed transforming into a normal seedling process of *Z. schneideriana* and provide a theoretical basis for improving the seed germination and seedling rate of *Z. schneideriana* and, at the same time, to improve scientific and technological support for the efficient use and protection of this species.

## 2. Materials and Methods

### 2.1. Plant Materials

*Z. schneideriana* seeds were harvested in mid-November 2022 from Duyun City, Guizhou Province, Southwest China (26°15′34″ N, 107°31′07″ E). In March 2023, six hundred seeds were washed with water, soaked in water solution for 24 h, and then sown in lime soil to facilitate seed germination. Seeds of four distinct developmental stages during the process of seed germination and subsequent seeding establishment were collected (60 seeds per stage, 240 seeds): A. Seeds soaked for 24 h; B. The seed coat ruptured nine days after sowing; C. Seven days after seed coat rupture, the embryos began to germinate; D. After 10 days of stage C, the seedlings were basically established. The samples were rinsed with distilled water. Subsequently, the samples from the four stages were promptly flash-frozen in liquid nitrogen and stored at −80 °C until further analysis. The samples were partitioned into two portions for metabolite (120 seeds) and total RNA extraction (120 seeds). The same sample criteria were used for both the transcriptome and metabolome assays: 30 seeds from each stage were determined and randomly divided into three bioreplication runs with 10 seeds per run.

### 2.2. Sample Metabolite Extraction

The sample (50 mg) was weighed, 1000 μL of the extract containing the inner target was swirled well for 30 s, and then, a steel ball was added, treated with a 45 Hz grinder for 10 min, followed by ultrasonic treatment for 10 min. After incubating at minus 20 °C for one hour, the sample was centrifuged at 4 °C for 12,000 rpm at 15 min. The 500 μL supernatant was taken out and put into the EP tube, the extract was dried in a vacuum concentrator, and 160 μL of the extract (acetonitrile water volume ratio: 1:1) was added to the dried metabolites for re-dissolution. The solution was swirled for 30 s and ultra-sonicated in an ice-water bath for 10 min. The samples were centrifuged at 12,000 rpm for 15 min at 4 °C. Finally, 120 μL of the supernatant was carefully taken out into a 2 mL sample bottle, and 10 μL of each sample was mixed into QC samples for machine testing.

### 2.3. LC-MS Analysis

The Waters Xevo G2-XS QTOF high-resolution mass spectrometer is capable of collecting primary and secondary mass spectrometry data in MSe mode, which is controlled by acquisition software (MassLynx V4.2, Waters, Germany). This instrument allows dual-channel data acquisition at low and high collision energies simultaneously. The specific parameter settings were as follows: the low collision energy is set at 2 V, while the high collision energy range is between 10 and 40 V, with a scanning frequency of 0.2 s for each mass spectrum. The electrospray ion source parameters were as follows: capillary voltage of 2000 V for positive ion mode or −1500 V for negative ion mode, cone voltage of 30 V, ion source temperature of 150 °C, desolvent gas temperature of 500 °C, backflush gas flow rate of 50 L/h, and desolventizing gas flow rate of 800 L/h. 

### 2.4. Data Preprocessing and Annotation and Metabolite Data Analysis

The raw data were processed using Progenesis QI v3.0 software for peak extraction, peak alignment, and other data-processing operations. The Progenesis QI software online METLIN database and Biomark’s self-built library were used for metabolite quantification and identification.

After normalizing the original peak area information to the total peak area, a follow-up analysis was performed. Principal component analysis and Spearman correlation analysis were used to assess the repeatability of the samples within the group and quality control samples. The identified compounds were searched for classification and pathway information in the KEGG databases. The method of combining the difference multiple, *p* value, and VIP value of the OPLS-DA model was adopted to screen the differential metabolites. The screening criteria were FC > 1, *p*-value < 0.05, and VIP > 1. The differences in metabolites with KEGG pathway enrichment significance were calculated using the hypergeometric distribution test.

### 2.5. Transcriptome Sequencing and De Novo Assembly

Total RNA was extracted using the RNAprep Pure Plant Kit (TIANGEN, Beijing, China), according to the manufacturer’s instructions. The extracted RNA was identified using 1% agarose gel electrophoresis. The purity, concentration, and integrity of RNA samples were tested using advanced molecular biology equipment to ensure the use of qualified samples for transcriptome sequencing. The purity and concentration of RNA were determined using a Nano Drop 2000 spectrophotometer (Thermo, Waltham, MA, USA), whereas RNA integrity was assessed using an Agilent 2100 LabChip GX (Santa Clara, CA, USA). After qualifying the samples, a cDNA library was constructed. Libraries were sequenced using the Illumina HiSeq 2000 platform. Subsequently, high-quality clean data were obtained by eliminating reads containing splices and low-quality reads from the library. Trinity was employed for de novo assembly of sequencing data [38]. The assembled transcripts were clustered to identify the longest transcripts as unigenes for subsequent analysis.

### 2.6. Functional Annotation and Differential Expression Analysis of Differentially Expressed Genes (DEGs)

DIAMOND [39] was used to perform comparative analysis between unigenes and the following databases: NCBI non-redundant protein sequences (NR), Protein family (Pfam), Clusters of Orthologous Groups (COG), euKaryotic Orthologous Groups (KOG), Evolutionary genealogy of genes: Non-supervised Orthologous Groups (Egg-NOG), Swiss-Prot Protein Sequence Database (Swiss-Prot), KEGG, and Gene Ontology (GO). Gene expression levels were estimated using RSEM [40] for each sample. The sequenced reads were aligned to the unigene library using Bowtie [41], expression levels were estimated by integrating RSEM [42], and the FPKM value was utilized to quantify the expression abundance of the corresponding unigene. Differential expression analysis of the two conditions/groups was performed using the DESeq R package (1.10.1). The thresholds for significantly different expression were set as q < 0.05 and |log2FoldChange| > 1. Enrichment analyses of GO and KEGG pathways in DEGs were performed using the GOseq R package (v1.10.0) and KOBAS (v2.0.12) [43].

### 2.7. Validation of Gene Expression Profiles through RT-qPCR

The expression levels of the nine DEGs were validated using RT-qPCR to confirm the results obtained from RNA-seq analysis. Primers were designed using Primer Premier software (5.0) (Table S1). Triplicate RT-qPCR reactions were performed using the Talent qPCR PreMix (SYBR Green) Kit (Tiangen, Beijing, China) on a CFX96 Touch Real-Time PCR System (Bio-Rad, Hercules, CA, USA) following the manufacturer’s instructions. The relative transcript levels for each gene were determined using the cycle threshold (Ct) and 2^−∆∆^Ct methods and normalized to UBC as an internal control.

### 2.8. Statistical Analysis

Statistical analysis of samples from the four stages and RT-qPCR data were performed using the SPSS 27 (Chicago, IL, USA). Analyses of variance (ANOVA) for sets of data were analyzed with Duncan’s test method to determine differences between pairs of means of multiple experiments; *p* < 0.05 and *p* < 0.01 were considered to be significant and extremely significant, respectively. Finally, TB tools, Origin 2022, and Adobe Photoshop 2023 (USA) were employed to generate diagrams and enhance the images.

## 3. Results

### 3.1. Transcriptome and Metabolome Functional Annotation and Expression Profiling

**Figure 1:**
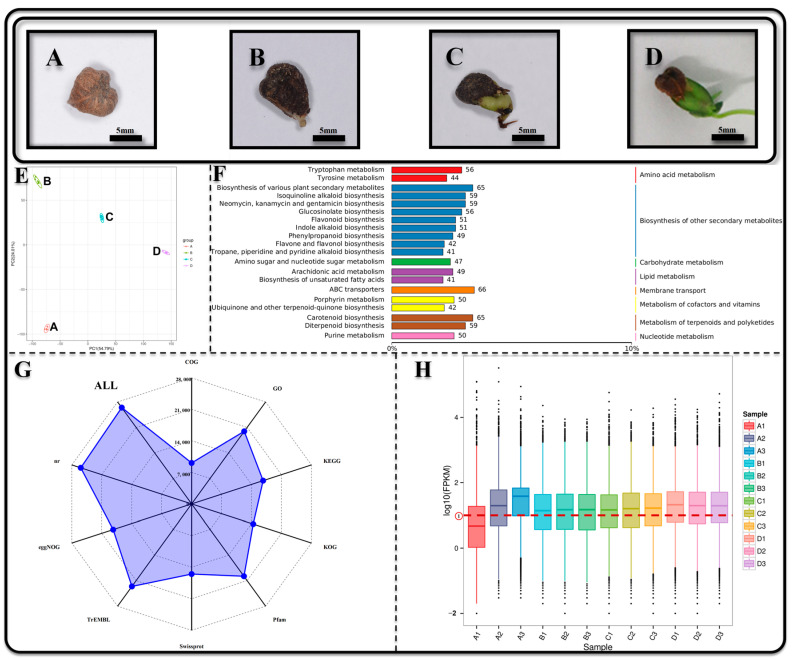
The functional annotation and expression profiling of metabolites and transcriptomes during distinct stages of seed germination to seeding establishment in *Zelkova schneideriana*. (**A**) Seeds undergo swelling after being immersed in a GA3 solution for 24 h; (**B**) the seed coat is breached and emergence of the radicle; (**C**) cotyledon begins to develop; (**D**) seedling establishment; (**E**) PCA analysis is conducted to examine various metabolites; (**F**) KEGG enrichment results show 20 metabolic pathways with the most types of metabolites; (**G**) the quantity of genes is annotated in different databases; (**H**) box plots illustrating FPKM values for each sample, and the red line represents the standard line with log10(FPKM) = 1.

Seeds transformed into a normal *Z. schneideriana* seedling process were categorized into four distinct stages with discernible morphological disparities: A, B, C, and D (Figure 1A–D). A and B were the stage of seed germination, and C and D were the stage of seedling establishment.

In this study, 5845 metabolites were detected in various samples (including 3002 metabolites in the positive ion mode and 2843 metabolites in the negative ion mode). KEGG enrichment analysis showed the 20 metabolic pathways with the most types of metabolites in positive and negative ion modes. These pathways were further categorized into seven groups, with the biosynthesis of secondary metabolites, lipid metabolism, and amino acid metabolism exhibiting the greatest number of metabolic pathways (Figure 1F). KEGG enrichment analysis revealed that the top 20 metabolic pathways were enriched with metabolites, which were further categorized into distinct groups (Figure 1F). A total of 12 cDNA libraries were constructed and sequenced, resulting in an acquisition of 75.85 Gb of high-quality sequencing data. The clean data obtained from each sample reached 5.84 Gb, with a Q30 base percentage exceeding 91.28% (Table S2). A total of 29,831 unigenes were obtained following assembly. Functional annotation was performed on the unigene dataset, resulting in 26,431 annotated unigenes obtained by comparing multiple databases (Figure 1G). The sequencing reads were compared to the unigene library, which revealed that the gene expression levels were predominantly high in most samples (log10FPKM > 1) (Figure 1H).

### 3.2. The Identification of Differentially Express Genes

**Figure 2:**
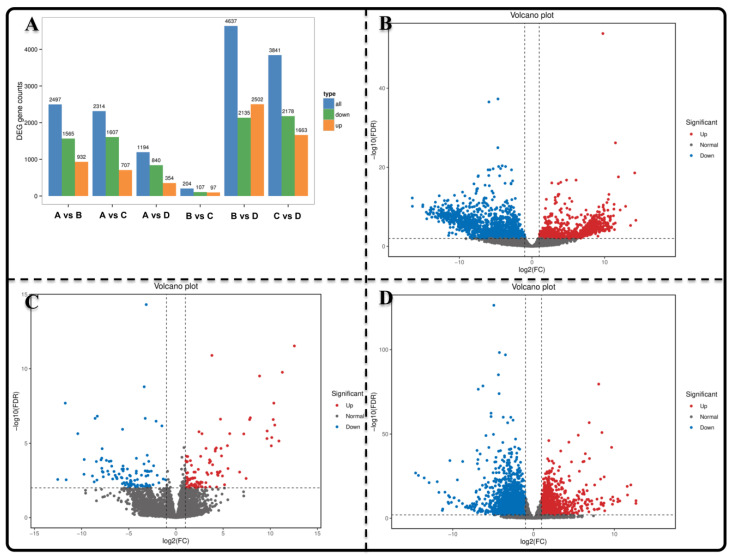
Analysis of the DEMs of *Z. schneideriana*. (**A**) Histogram depicting statistics of DEGs; (**B**–**D**) Volcano plots illustrating DEGs expression in AB, BC and DC groups.

All clean reads from each sample were mapped to the assembled unigenes to analyze differential gene expression levels during various seed germination stages until seeding establishment. A substantial number of DEGs with significant variations in their distribution patterns were identified during the transformation of seeds into normal seedlings (Figure 2A–D). In group AB, there were a total of 2497 DEGs (932 upregulated and 1565 downregulated), while group BC had 204 DEGs (97 upregulated and 107 downregulated), and group DC exhibited the highest number of 3641 DEGs (1663 upregulated and 2178 downregulated). 

### 3.3. GO and KEGG Enrichment Analysis of Differentially Expressed Genes

**Figure 3:**
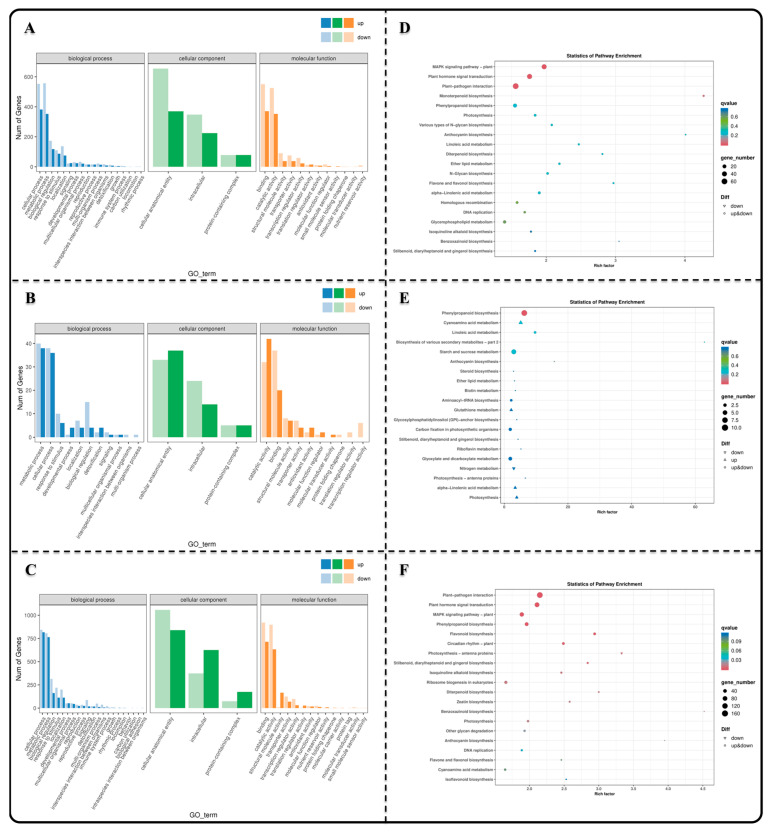
GO and KEGG enrichment analysis of DEGs. (**A**–**C**) Taxonomic maps with GO annotations showing DEGs in AB, BC and DC groups; (**D**–**F**) KEGG enrichment bubble maps displaying the top 20 pathways enriched with DEGs expression in AB, BC and DC groups.

GO and KEGG enrichment analyses were carried out to elucidate the gene regulatory networks and key regulatory genes that play a role in the transformation of seedling formation. The GO enrichment analysis revealed that the majority of DEGs across the different comparison groups were significantly enriched in the cellular process (GO:0009987), metabolic process (GO:0006807), cellular anatomical entity (GO:0110165), and binding (GO:0019899) and catalytic activity (GO:0003824) (Figure 3A–C). Furthermore, the KEGG enrichment analysis demonstrated that Group AB was predominantly associated with the MAPK signaling pathway plant (ko04016), plant hormone signal transduction (ko04075), plant–pathogen interaction (ko04626), monoterpenoid biosynthesis (ko00261), and phenylpropanoid biosynthesis (ko00940) (Figure 3D). The BC group primarily showed enrichment in the phenylpropanoid biosynthesis, cyanoamino acid metabolism (ko00460), linoleic acid metabolism (ko00591), biosynthesis of various plant secondary metabolites (ko00999), and starch and sucrose metabolism (ko00500) (Figure 3E). On the other hand, the DC group mainly exhibited enrichment in the plant–pathogen interaction, plant hormone signal transduction, MAPK signaling pathway, phenylpropanoid biosynthesis, and flavonoid biosynthesis pathways (ko00941) (Figure 3F). The enrichment results suggested that the regulation of seed germination and seeding establishment was strongly dependent on the transduction of intraseed signaling molecules.

### 3.4. The Identification and KEGG Enrichment Analysis of Differentially Expressed Metabolites (DEMs)

**Figure 4:**
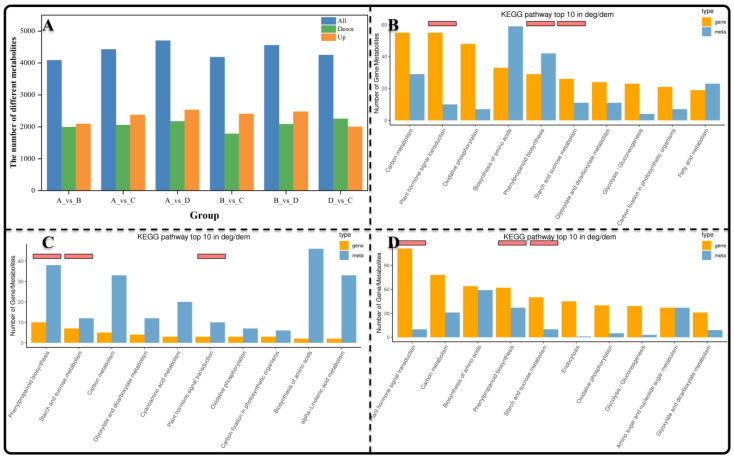
Analysis of DEMs and co-enrichment results of KEGG of DEGs and DEMs. (**A**) Statistical histogram of DEMs; (**B**–**D**) The top 10 pathways with the highest number of DEGs and DEMs in the AB, BC and CD groups after KEGG enrichment.

A significant number of DEMs were identified in this study (Table S3). A total of 4084 DEMs (1900 downregulated and 2094 upregulated) were identified in group AB (Figure 4A). In the BC group, there were 4185 DEMs (1781 downregulated and 2404 upregulated). The DC group contained 4250 DEMs (2249 downregulated and 2001 upregulated). Subsequently, we performed a KEGG joint analysis of DEGs and DEMs, revealing the top ten metabolic pathways enriched with the highest number of DEGs and DEMs in the AB, BC, and DC groups (Figure 4B–D). The top five metabolic pathways identified in group AB included carbon metabolism, plant hormone signal transduction, oxidative phosphorylation, amino acid biosynthesis, and phenylpropanoid biosynthesis (Figure 4B). The top five metabolic pathways identified in group BC were phenylpropanoid biosynthesis, starch and sucrose metabolism, carbon metabolism (ko01200), glyoxylate and dicarboxylate metabolism (ko00630), and cyanoamino acid metabolism (Figure 4C). The top five metabolic pathways identified in the DC group were plant hormone signal transduction, carbon metabolism, biosynthesis of amino acids (ko01230), phenylpropanoid biosynthesis, and starch and sucrose metabolism (Figure 4D). Notably, the most significantly enriched metabolic pathways across different comparison groups included plant hormone signal transduction, carbon metabolism, amino acid biosynthesis, phenylpropanoid biosynthesis, and starch and sucrose metabolism. These metabolic pathways have a profound influence on plant growth. To investigate the mechanisms underlying material metabolism and gene regulation during seed germination and seeding establishment, we focused on the plant hormone signal transduction, starch and sucrose metabolism, linoleic acid metabolism, and phenylpropanoid biosynthesis.

### 3.5. Seed Germination and Seeding Establishment Are Regulated by Hormone Signals

**Figure 5:**
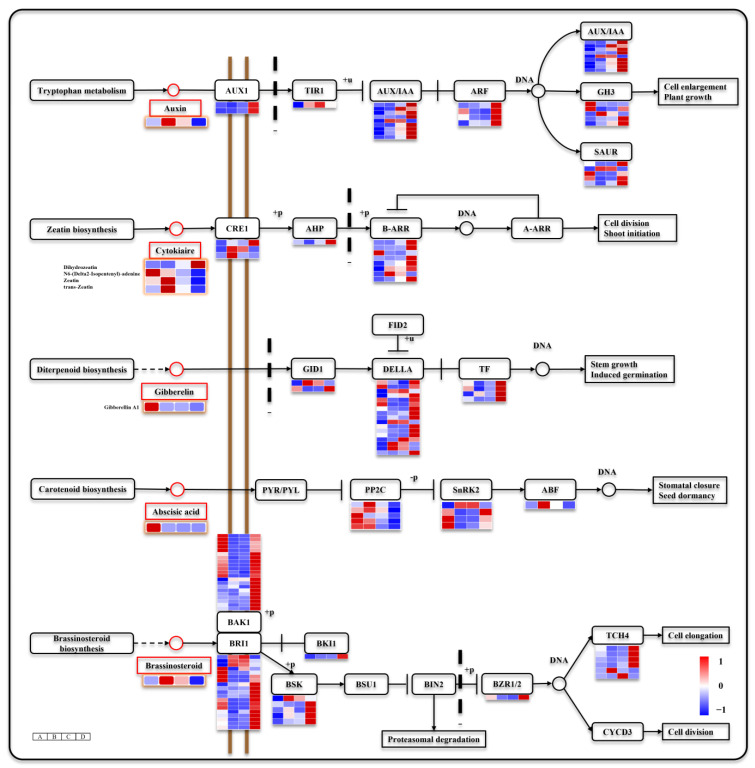
Plant hormone signal transduction. Note: The heat map with orange circles represents changes in metabolite metabolism levels at different stages. The heat map in the figure shows four periods A–D from left to right.

**Figure 6:**
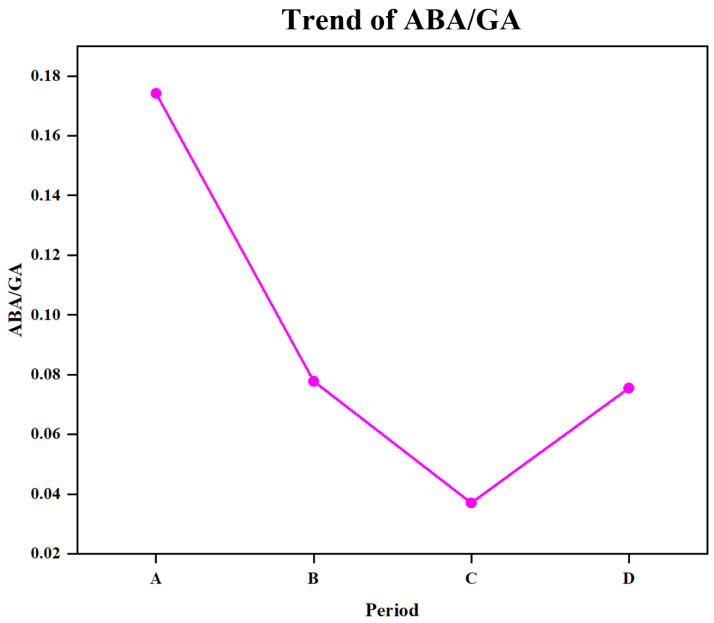
Line chart of ABA/GA trends.

Plant hormones regulate seed germination and seeding establishment. During seed germination and seeding establishment, 168 DEGs were enriched in hormone signaling pathways, and we focused on the auxin, cytokiaire, gibberellin, abscisic acid, and brassinolide signaling pathways (Figure 5). In these metabolic pathways, IAA, CTKs, GA1, ABA, and BR contents were significantly different at different germination stages. Among them, four CTKs were detected in both positive and negative ion modes: Dihydrozeatin (DHZ), N6-(Delta2-Isopentenyl)-adenine, zeatin (ZT), and trans-zeatin (TZT). N6-(Delta2-Isopentenyl)-adenine exhibited a high metabolic level during stage A; the metabolic levels of ZT and TZT increased during stage B but decreased during stages C and D; the metabolic level of DHZ increased during stage D. Different CTKs might have exerted distinct functions across different stages. The ABA and GA1 contents were highest at stage A and then significantly decreased. IAA and BR were highest at stage B and gradually decreased at stage C and D. In addition, ABA/GA was significantly reduced after release from dormancy (Figure 6). Most DEGs were found in the signaling pathways of these hormones. In the auxin metabolic pathways, DEGs include *auxin influx carrier* (*AUX1*), *transport inhibitor response 1* (*TIR1*), *auxin-responsive protein IAA* (*AUX/IAA*), *auxin response factor* (*ARF*), auxin *responsive GH3 gene family* (*GH3*), and *SAUR family protein* (*SAUR*). In cytokinin signaling pathways, DEGs include *Arabidopsis histidine kinase* (*CRE1*, EC:2.7.13.3), *histidine-containing phosphotransfer protein* (*AHP*), and *two-component response regulator ARR-B family* (*B-ARR*). In gibberellin signaling pathways, DEGs include *gibberellin receptor GID1* (*GID1*), *DELLA protein* (*DELLA*), and *phytochrome-interacting factor* (*TF*). In abscisic acid signaling pathways, DEGs include *protein phosphatase 2C* (*PP2C*, EC:3.1.3.16), serine/threonine-protein kinase SRK2 (SnRK2, EC:2.7.11.1), and *ABA responsive element binding factor* (*ABF*). In brassinolide signaling pathways, DEGs include *brassinosteroid insensitive 1-associated receptor kinase 1* (*BAK1*, EC:2.7.10.1 2.7.11.1), *protein brassinosteroid insensitive 1* (*BRI1*, EC:2.7.10.1 2.7.11.1), *BRI1 kinase inhibitor 1* (*BKI1*), *BR-signaling kinase* (*BSK*, EC:2.7.11.1), *brassinosteroid resistant 1/2* (*BZR1/2*), and *xyloglucan: xyloglucosyl transferase TCH4* (*TCH4*, EC:2.4.1.207). It is worth noting that most DEGs in the abscisic acid signaling pathway were highly expressed at stage A, and ABA signaling may be mainly activated during seed dormancy. Conversely, most DEGs in the auxin, cytokinin, gibberellin, and brassinolide signaling pathways were upregulated at stage D, and they may mainly regulate seeding establishment. 

### 3.6. Releasing of Stored Carbohydrates during Seed Germination and Seeding Establishment

**Figure 7:**
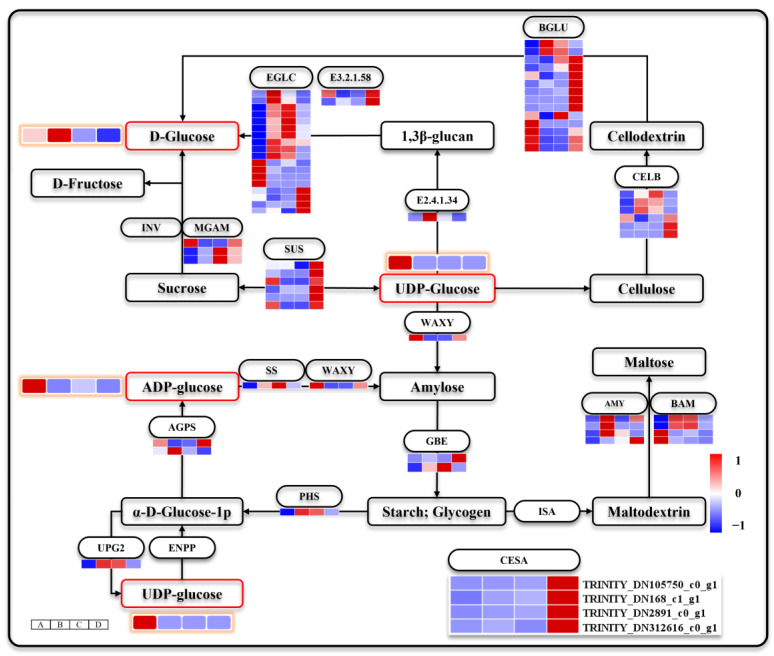
Starch and sucrose metabolism. Note: The heat map with orange circles represents changes in metabolite metabolism levels at different stages. The heat map in the figure shows four periods A–D from left to right.

Seed germination and seeding establishment require a continuous supply of energy and material. The sucrose and starch metabolic pathways ensure uninterrupted provision of hexoses for subsequent metabolism. Ninety DEGs were enriched in the metabolic pathways of sucrose and starch (Figure 7). The levels of D-glucose, UDP-glucose, and ADP-glucose exhibited significant variations across different time stages. ADP-glucose and UDP-glucose peaked during stage A before gradually declining, while D-glucose increased after stage A but progressively decreased during stages C and D. Transcriptomic analysis revealed distinct characteristics of sugar metabolism during different stages. D-glucose serves as a crucial source of material and energy in plants, and the expression patterns of DEGs involved in its regulation vary across these stages. *maltase-glucoamylase* (*MGAM*, EC:3.2.1.20 3.2.1.3) exhibited predominant upregulation in stages C and D, while *glucan en-do-1,3-beta-D-glucosidase* (*EGLC*, EC:3.2.1.39) and *glucan 1,3-beta-glucosidase* (*E2.4.1.34*, EC:3.2.1.58) were mainly upregulated in stages B and C. *beta-glucosidase* (*BGLU*, EC:3.2.1.21) showed significant upregulation in stages A and D, whereas *endoglucanase* (*CELB*, EC:3.2.1.4) was primarily upregulated in stages B, C and D. Notably, DEGs regulating starch metabolism (*Soluble starch synthase* (*SS*, EC:2.4.1.21), *glycogen phosphorylase* (*PHS*, EC:2.4.1.1), *α-amylase* (*AMY*, EC:3.2.1.1), and *β-amylase* (*BAM*, EC:3.2.1.2)) were prominently upregulated during stages B and C. These stage-specific upregulated sugar metabolic pathways may adapt to varying levels of D- glucose supply required for seed germination and seeding establishment by modulating the utilization rate of sugars. During the cellulose chain elongation stage, UDP-glucose acts as a substrate for cellulose synthase (CESA) to add glucose groups to the cellulose chain. SUS catalyzes the synthesis of UDP-glucose along with the UTP-glucose-1-phosphate uridylyl transferase (UPG2, EC:2.7.7.9). It is worth mentioning that all DEGs encoding *Sucrose synthase* (*SUS*, EC:2.4.1.13) and *CESA* were significantly upregulated during stage D. However, UPG2 was found to be upregulated during stages B and C as well. The pathway supplying UDP-glucose may also affect the seed germination and seeding establishment.

### 3.7. Linoleic Acid Metabolism Is Mainly Activated after Seed Germination

**Figure 8:**
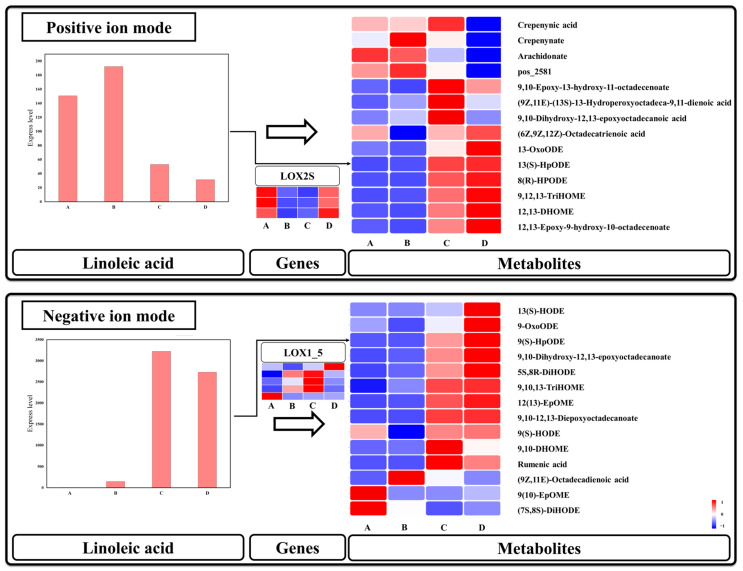
Linoleic acid metabolism. Note: The heat map in the figure shows four periods A–D from left to right.

Linoleic acid metabolism involves a series of catalytic reactions with linoleic acid as the substrate and plays a pivotal role in plant growth, development, and stress resistance. In this metabolic pathway, eight DEGs encoding these two enzymes were identified (Figure 8). Interestingly, *lipoxygenase* (*LOX2S*, EC:1.13.11.12) displayed an upregulated expression pattern during stages A and D, while *linoleate 9S-lipoxygenase* (*LOX1_5*, EC:1.13.11.58) showed predominant expression during stages B and C. Although only a few genes were enriched by KEGG analysis, numerous metabolites were detected. The LC-MS instrument offers two determination modes, namely positive ion mode and negative ion mode, for metabolite analysis. In general, the positive ion mode facilitates the determination of alkaline compounds with higher accuracy, whereas the negative ion mode enables more accurate determination of acidic compounds. Specifically, in the positive ion mode, the level of linoleic acid metabolism increased at stage B but decreased at stages C and D. Conversely, in the negative ion mode, there was a significant increase in linoleic acid metabolism at stages C and D. Notably, the metabolic level of linoleic acid was considerably higher in the negative ion mode than in the positive ion mode. Moreover, considering that linoleic acid is an acidic compound, detection results obtained in the negative ion mode may provide more accurate information. Across the different modes of detection, most metabolites derived from linoleic acid exhibited sustained high levels during stages C and D. Based on these findings, we infer that the activation of linoleic acid metabolism primarily occurs after seed germination and plays a crucial role in seeding establishment.

### 3.8. Phenylpropane Biosynthesis Is also Involved in Seed Germination and Seeding Establishment

**Figure 9:**
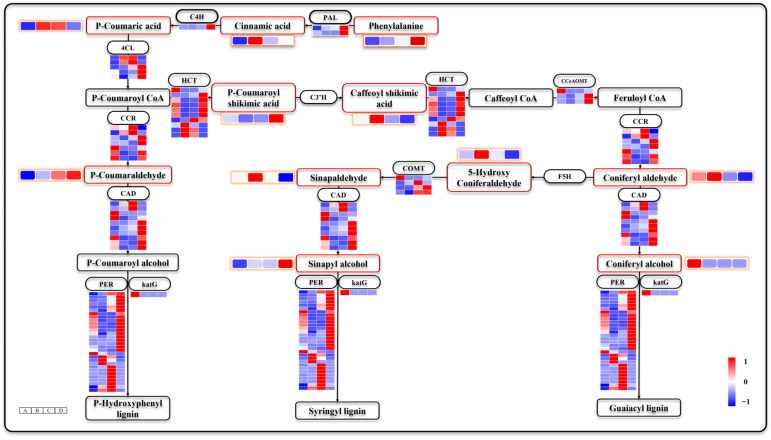
Note: The heat map with orange circles represents changes in metabolite metabolism levels at different stages. The heat map in the figure shows four periods A–D from left to right.

Lignin, a crucial constituent of plant roots and stems, is synthesized via the phenylpropanoid biosynthesis [44]. In this study, we identified 93 DEGs that were enriched in the phenylpropanoid biosynthesis pathway (Figure 9). The levels of intermediates, including phenylalanine, trans-cinnamate, p-coumaric acid, caffeoyl shikimic acid, p-coumaraldehyde, sinapaldehyde, 5-hydroxy coniferaldehyde, and coniferyl aldehyde, exhibited an increasing trend from stages A to B, followed by a gradual decrease in stages C and D. Moreover, the levels of phenylalanine, p-coumaroyl shikimic acid, p-coumaraldehyde, and sinapyl alcohol showed an overall increase across all stages A–D. Sinapyl alcohol and coniferyl alcohol are the precursors of S-lignin and G-lignin, respectively. Notably, coniferyl alcohol levels remained low after stage A. These findings suggest that lignin monomers required for seed germination and seeding establishment may differ. Transcriptomic analysis revealed differential expression patterns of several key genes involved in the phenylpropanoid biosynthesis. DEGs includes *phenylalanine ammonialyase* (*PAL*, EC:4.3.1.24), *cinnamate 4-hydroxylase* (*C4H*, EC:1.14.14.91), *4-coumarate: CoA ligase* (*4CL*, EC:6.2.1.12), *hydroxycinnamoyl-CoA* (*HCT*, EC:2.3.1.133), *caffeoyl-CoA O-methyl transferase* (*CCoAOMT*, EC:2.1.1.104), *cinnamoyl-CoA reductase* (*CCR*, EC:1.2.1.44), *cinnamyl alcohol dehydrogenase* (*CAD*, EC:1.1.1.195), *caffeic acid O-methyltransferase* (*COMT*, EC:2.1.1.68 2.1.1.4), *peroxidase* (*PER*, EC:1.11.1.7), and *peroxidase* (*katG*, EC:1.11.1.21). Among them, *PAL* and *C4H* maintained high expression, specifically in stage D, suggesting distinct metabolic requirements during seed germination and seeding establishment.

### 3.9. Quantitative Real-Time PCR (RT-qPCR) Validation

**Figure 10:**
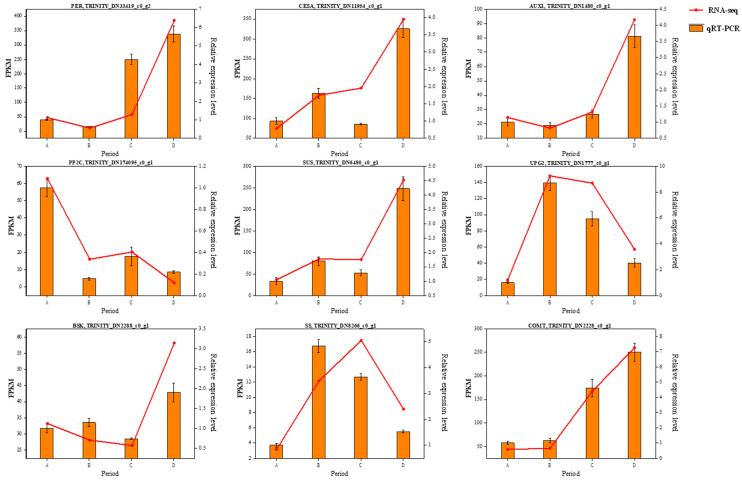
Relative expression via qRT-PCR and RNA-seq of nine DEGs. The heat map in the figure shows four periods A–D from left to right.

To validate the transcriptome data, nine DEGs were randomly selected for expression analysis by RT-qPCR. These DEGs exhibited consistent expression patterns between RNA-seq and RT-qPCR, providing evidence of the reliability of Illumina sequencing (Figure 10).

## 4. Discussion

### 4.1. ABA and GA Are Key Hormones Involved in Seed Germination and Affect the Synthesis of Other Hormones

Previous studies have demonstrated a significant decrease in ABA signaling during seed maturation, accompanied by an increase in GA signaling, ultimately leading to seed germination [45]. Abundant evidence has shown that ABA plays a crucial role in seed dormancy and inhibits plant seed germination with a reduction in ABA content, which serves as an important marker for releasing seed dormancy [46,47]. In this study, beech seeds were released from dormancy and germinated after immersion in a GA3 solution under suitable conditions. During germination, ABA content remained high only during stage A but significantly decreased throughout the B, C, and D stage. The ABA/GA ratio is considered to be a key factor influencing seed germination [14]. Although the exogenous application of GA3 was instrumental in releasing beech seed dormancy, it is intriguing that even after its release, the calculated ratio of these two hormones (ABA/GA) continued to decrease significantly (Figure 6). In contrast to ABA, the levels of IAA, ZT, TZT, and BR increase once cellular development commences. Previous studies have revealed the inhibitory effect of ABA on IAA and BR synthesis, as well as antagonistic interactions between ABA and CTK signaling pathways [48,49,50]. We propose that ABA suppresses IAA, CTKs, and BR biosynthesis in *Z. schneideriana* seeds. As dormancy gradually subsided, there was a decline in ABA content, along with relief from inhibition. Consequently, signal transduction mediated by auxin, cytokinin, and brassinolide becomes progressively activated because of their increasing levels. However, their accumulation subsequently decreased during stages B, C, and D, indicating their utilization following involvement in root and leaf development.

*PP2C* and *SnRK* are pivotal genes in the regulation of seed dormancy [51]. At low ABA levels, PP2C binds to SnRK to deactivate the downstream ABA signaling factors. Transcriptomic analysis revealed that even after a decrease in ABA content at Stage B, *PP2C* remained highly expressed (Figure 4), thereby impeding the transmission of ABA signaling. Moreover, DELLA inhibits GA signaling and promotes ABA accumulation in seeds; endosperms lacking DELLA release less ABA [52,53]. In this study, most *DELLA* were found to be transcriptionally downregulated at stage A, which facilitated GA function but hindered DELLA accumulation, consequently suppressing ABA accumulation. Notably, most DEGs involved in IAA, CTKs, GA, and BR signaling were significantly upregulated at stage D, with a particular emphasis on BR. This leads to the activation of multiple hormone-mediated metabolic pathways and mobilization of complex regulatory networks essential for plant development. In summary, our findings suggest that multiple hormones participate in regulating seed germination, and previous studies have highlighted their crucial roles in both seed germination and plant growth [8].

### 4.2. Seed Germination and Seeding Establishment Have Different Characteristics of Sugar Metabolism

When seeds break dormancy, a substantial amount of sugar reserves in the endosperm are mobilized to participate in the intricate metabolic processes associated with seed germination. UDP-glucose is an indispensable substrate for sucrose and cell wall synthesis [54]. ADP-glucose directly acts as a precursor for starch synthesis and is converted into starch through catalysis by ADP-glucose pyrophosphorylase [55]. The levels of UDP-glucose and ADP-glucose reserves in seeds significantly declined after the completion of stage A, indicating their crucial role in seed coat rupture, along with the concurrent consumption of starch and sucrose. Starch is the primary reserve compound accumulated over an extended stage until seed germination [55], and hydrolysis of starch granules has also been observed during wheat seed germination [56]. The release of starch and sucrose signifies the activation of sugar metabolism upon the release of seed dormancy. In contrast, D-glucose accumulated in stage B. D-glucose is the source of energy for cell function, and the regulation of its metabolism is of great importance [57]. Of course, we believe that D-glucose is consumed continuously, and we also infer that the accumulation of D-glucose is more conducive to the sustained growth of plants after seed coat breakthrough.

Corresponding to the decrease in ADP-glucose and UDP-glucose reserves, most DEGs encoding *SS*, *GBE*, *PHS*, *AMY*, *BAM*, *UPG2*, *E2.4.1.34*, *EGLC*, and *CELB* were activated after being released from dormancy. These DEGs constitute the complete metabolic pathway of starch released into D-glucose, which may be essential for starch release, cellulose formation, and energy and substance metabolism during seed germination in *Z. schneideriana*. Notably, all DEGs encoding *MGAM*, *SUS*, and *CESA* were significantly upregulated at stage D. Sucrose is the main transport form of sugar in plants [58], and it has a higher metabolic rate than starch. Unlike seed coat breakthroughs, seeding establishment requires more intense substance and energy metabolisms. We speculated that the sucrose decomposition process regulated by *MGAM* may be more beneficial for the formation of D-glucose after seed germination. UDP-glucose is essential for cell-wall biosynthesis [54]. SUS catalyzes the reversible transformation of UDP-glucose into sucrose [59], which converts sucrose into UDP-glucose when UDP-glucose levels are low. The supply of UDP-glucose provides CESA with the material basis for the continuous formation of cellulose, which is important for seeding establishment of *Z. schneideriana*.

### 4.3. Lipids Release May Serve as a Potential Supplement in the Case of Sugar Deficiency

Lipids hydrolysis generates glycerol and fatty acids, which are oxidized under a sufficient oxygen supply to provide energy and substrates for the initial growth of seedlings [60]. Linoleic acid, one of the main unsaturated fatty acids found in seeds, shows an increase in its content as a sign of released stored lipids. In this study, the content of linoleic acid and its derivatives began to increase at stage B and significantly increased at stages C and D, suggesting that the released lipids may assist in seed germination to some extent but are primarily utilized during plant establishment. Previous studies have demonstrated that the utilization of stored lipids begins during dormancy lifting and germination [61,62], supporting our conclusion. Interestingly, unlike the significant increase in the levels of linoleic acid and its derivatives during seedling establishment, the sugar metabolism levels decreased at this stage. As seeds break through their coat, there is increased absorption of oxygen, leading to accelerated oxidation of fatty acids to release more energy. With starch being consumed in large amounts, further oxidation of fatty acids produced by lipid release may supplement the energy required for seedling establishment when the sugar supply is insufficient. Previously, it was discovered that starch and sucrose pathways were significantly upregulated during seed germination in *Polygonatum cyrtonema* [63]. Similarly, in *Cinnamomum migao*, genes related to carbohydrate and lipid metabolism were predominantly activated after seed coat rupture [64]. Additionally, there was a decrease in total starch and crude oil content during seed germination of *Phyllostachys edulis*, whereas genes involved in starch metabolism and lipid body degradation pathways were consistently upregulated [65]. These findings align with our own results; however, unlike these studies, we inferred a relationship between starch and lipid release based on our results. 

Transport and degradation of stored lipids is an important physiological and biochemical metabolic process during plant seed germination, with LOX located on lipid body membranes being rate-limiting enzymes [66]. In this study, *LOX1_5* was upregulated in stages B and C, while most *LOX2S* were upregulated in stages A/D, suggesting roles mainly in seed germination or regulation of linoleic acid oxidation. Furthermore, LOX has been demonstrated to contribute to the biosynthesis of jasmonic acid (JA) and is implicated in various crucial biological processes, including conferring resistance to external abiotic stresses, insects, and pathogens [67].

### 4.4. The Formation of G-Lignin and S-Lignin Contributes Differently to Seed Germination and Seeding Establishment

Lignin monomers, which constitute the primary constituents of the secondary cell wall in plants, are synthesized via the phenylpropanoid pathway [68]. In angiosperms, lignin is predominantly formed by the polymerization of G-lignin and S-lignin [69]. The significant enrichment of numerous DEGs and DEMs in the phenylpropanoid pathway suggests that lignin biosynthesis plays a crucial role in seed germination and seeding establishment of *Z. schneideriana*. Previous studies on seed germination in other plants have highlighted the involvement of the phenylpropanoid pathway [25,42]. In this study, most metabolites within the phenylpropanoid pathway exhibited increased levels after stage A, indicating that phenylpropanoid metabolism may be primarily activated following dormancy release. However, it is noteworthy that the coniferoyl alcohol content was initially high during stage A but significantly decreased during stage B. As a precursor to G-lignin synthesis, the consumption of coniferoyl alcohol inevitably leads to an increase in G-lignin production during seed germination. The accumulation of lignin within the secondary cell wall promotes enhanced mechanical strength in cells [70]. By increasing radicle deposition through enhanced mechanical strength, G-lignin may facilitate seed coat rupture and germination completion. Sinapyl alcohols, as precursors to S-lignin, were found at low levels during stage A, but gradually increased from stage A to stage D. Therefore, it can be inferred that S-lignin deposition mainly occurs after germination.

Vascular tissues perform crucial functions in transporting substances, such as water, inorganic salts, and photosynthetic products, while providing mechanical support to plants [71]. The establishment of roots, stems, and leaves after seed germination requires significant lignin deposition for the construction of new vascular tissues. Transcriptomic analysis revealed that most DEGs involved in the phenylpropanoid biosynthesis pathway were activated during stage D. PAL, which is considered a rate-limiting enzyme in phenolic compound synthesis, serves as the first enzyme in this metabolic pathway [72]. PAL’s transcription level reflects the intensity of phenylpropanoid biosynthesis; its significant upregulation during stage D indicates that this pathway primarily contributes to seeding establishment. Previous studies have also reported significantly increased PAL expression levels following buckwheat seed germination. *COMT* is a key regulator gene responsible for S-lignin formation, and inhibiting *COMT* expression leads to the loss of S-lignin synthesis [73]. *COMT* was predominantly activated during stage C and D, corresponding to sinapyl alcohol accumulation, indicating that S-lignin deposition mainly begins after seed germination. As G-lignin and S-lignin share coniferyl aldehyde as their precursor molecules, an increase in S-lignin synthesis may lead to decreased G-lignin production, potentially reflecting the biased selection of *Z. schneideriana* seeds to complete seed germination and establish plants.

## 5. Conclusions

**Figure 11:**
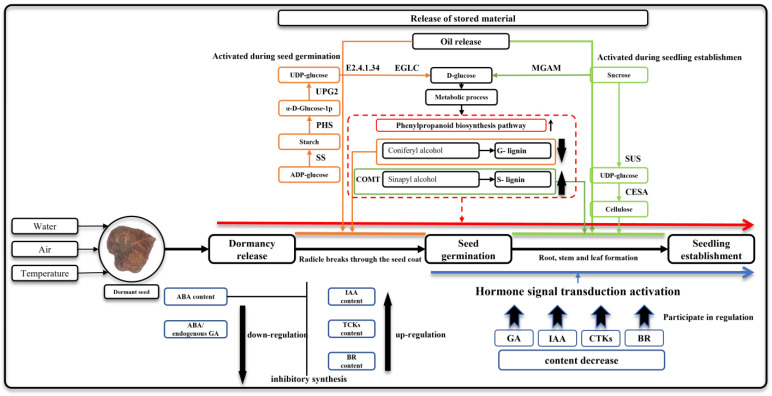
Hypothesized regulatory model for seed germination and seeding establishment in *Z. schneideriana*. Note: The substances and genes involved in the origin line mainly play a role in seed germination; the substances and genes involved in the green line mainly play a role in seedling establishment; the blue line reveals the characteristics of hormones that play a role; the red line indicates that phenylpropanoid biosynthesis is involved in seed germination and seedling establishment.

To elucidate the underlying regulatory mechanisms governing seed germination and seeding establishment in *Z. schneideriana*, we employed non-targeted metabolomic and transcriptomic analyses to investigate the genes and metabolic regulatory networks involved in these processes, ultimately constructing a putative regulatory model (Figure 11). The findings revealed the pivotal roles of hormone signal transduction, starch and sucrose metabolism, linoleic acid metabolism, and phenylpropanoid biosynthesis pathways during seed germination and seeding establishment. Further analysis demonstrated that exogenous GA3 application reduced ABA content and ABA/GA ratio, thereby breaking dormancy in *Z. schneideriana* seeds; subsequent release of ABA inhibition led to increased levels of IAA, TCK, and BR hormones. These hormones, together with GA, co-regulate the process of seeding establishment in *Z. schneideriana*. Under the influence of hormonal signaling, genes associated with starch metabolism and linoleic acid metabolism were activated to mobilize reserves such as starch, ADP-glucose, UDP-glucose, and lipids for participation in seed germination. During the seeding establishment phase, a large number of DEGs related to sucrose metabolism were upregulated to enhance the metabolic rate. Significantly, *CESA* expression was upregulated, and alterations were observed in the major pathway for D-glucose formation. Simultaneously, the rate of lipid decomposition may escalate to provide the plant with the requisite constituents and energy substrates essential for growth. Moreover, the activation of the phenylpropanoid metabolic pathway predominated during the seeding establishment stage, where G-lignin potentially reinforced the mechanical strength required for radicle penetration through the seed coat. Accelerated synthesis of S-lignin may play a crucial role in new vascular tissue formation. This study unraveled potential regulatory mechanisms underlying seed germination and seeding establishment in *Z. schneideriana*, which fills existing knowledge gaps and provides novel insights for related research endeavors.

## Data Availability

The transcriptome sequence data that support the findings of this study are openly available in GenBank of NCBI at (https://www.ncbi.nlm.nih.gov/ accessed on 5 March 2024)) under the accession no PRJNA1066040.

## Supplementary Materials

The following supporting information can be downloaded at: https://www.mdpi.com/article/10.3390/genes15040488/s1, Table S1: Primers for real-time fluorescence quantitative PCR. Table S2: Evaluation of sample sequencing data. Table S3: Differential metabolite statistics of the two modes.
