# Supplementary material for: Combined Analysis of Untargeted Metabolomics and Transcriptomics Revealed Seed Germination and Seedling Establishment in Zelkova schneideriana
Source: Genes (Basel). 2024 Apr 12;15(4):488. doi: 10.3390/genes15040488 (PMC11050531; doi:10.3390/genes15040488)
Supplement: Supplementary file 1 [file genes-15-00488-s001.zip › Supplementary material/Table.docx]

Table S1: Primers for real-time fluorescence quantitative PCR.

| **Gene ID** | **Gene annotation** | **Primer Sequence** |
| --- | --- | --- |
| TRINITY_DN33419_c0_g2 | *PER* | F: AGGAATGCCCTCAAACT |
|  |  | R: GAGAAAGCACCAACGAA |
| TRINITY_DN11954_c0_g1 | *CESA* | F: GCGATTGGGTTATGGATAG |
|  |  | R: GAGTGTGGGATGTCTCTGTTC |
| TRINITY_DN1480_c0_g1 | *AUX1* | F: CGTATGGGAGAAGGTGA |
|  |  | R: AACAGCGGAGTTTATGG |
| TRINITY_DN174095_c0_g1 | *PP2C* | F: GTGATGGAGGGGTGTTTT |
|  |  | R: CAGGTCTTTGGGGCTTA |
| TRINITY_DN6480_c0_g1 | *SUS* | F: TAAAGGACCGAAACAAACC |
|  |  | R: TCTCCAGCCACCACGAC |
| TRINITY_DN1777_c0_g1 | *UPG2* | F: AGACACCAGCAACAGATT |
|  |  | R: GTCAAGAGGGTTTTCACTA |
| TRINITY_DN2288_c0_g1 | *BSK* | F: CAATCTTCACGGGCACCA |
|  |  | R: CCGCTCTCGGAAACAATAAA |
| TRINITY_DN8266_c0_g1 | *SS* | F: AAAGCAACAAAGGGACA |
|  |  | R: CAATCTTATCAAGCAGCAA |
| TRINITY_DN2228_c0_g1 | *COMT* | F: ATGACGGTACAATGGCCTAAGGATC |
|  |  | R: GATCCTTAGGCCATTGTACCGTCAT |
| Reference gene | *UBC* | F: TCCTTCTATTGGGTTTG |
|  |  | R: CTGAGATGGGATGGTAA |

Table S2: Evaluation of sample sequencing data

| **SampleID** | **ReadSum** | **BaseSum** | **GC(%)** | **N(%)** | **Q20(%)** | **CycleQ20(%)** | **Q30(%)** |
| --- | --- | --- | --- | --- | --- | --- | --- |
| A1 | 21731514 | 6507502932 | 49.81 | 0.00 | 97.85 | 100.00 | 94.01 |
| A2 | 21445971 | 6388924208 | 50.10 | 0.00 | 97.45 | 100.00 | 93.45 |
| A3 | 20099534 | 5992448241 | 47.81 | 0.00 | 97.09 | 100.00 | 92.96 |
| B1 | 22095123 | 6616437971 | 45.84 | 0.01 | 97.63 | 100.00 | 93.86 |
| B2 | 20508120 | 6138827682 | 45.53 | 0.00 | 97.81 | 100.00 | 93.80 |
| B3 | 20533586 | 6145384166 | 45.30 | 0.00 | 97.86 | 100.00 | 93.91 |
| C1 | 19720866 | 5903403096 | 46.30 | 0.00 | 97.54 | 100.00 | 93.56 |
| C2 | 19815920 | 5933084274 | 45.31 | 0.00 | 98.09 | 100.00 | 94.37 |
| C3 | 20978382 | 6280784974 | 45.57 | 0.00 | 97.96 | 100.00 | 94.10 |
| D1 | 23476459 | 7027841614 | 45.69 | 0.00 | 96.51 | 100.00 | 91.28 |
| D2 | 23626872 | 7072883134 | 45.41 | 0.00 | 97.78 | 100.00 | 93.90 |
| D3 | 19516651 | 5842346808 | 46.02 | 0.00 | 97.45 | 100.00 | 93.19 |

Table S3: Differential metabolite statistics of the two modes

| **Group** | **Total_num** | **Diff_num** | **Up_num** | **Down_num** |
| --- | --- | --- | --- | --- |
| D_vs_C | 5845 | 4250 | 2249 | 2001 |
| B_vs_D | 5845 | 4558 | 2083 | 2475 |
| B_vs_C | 5845 | 4185 | 1781 | 2404 |
| A_vs_D | 5845 | 4701 | 2171 | 2530 |
| A_vs_B | 5845 | 4084 | 1990 | 2094 |
| A_vs_C | 5845 | 4425 | 2053 | 2372 |
